# Untargeted Metabolomics Combined with Solid Phase Fractionation for Systematic Characterization of Bioactive Compounds in Hemp with Methane Mitigation Potential

**DOI:** 10.3390/metabo12010077

**Published:** 2022-01-13

**Authors:** Rikke Hald Jensen, Marie Rønn, Mirka Thorsteinsson, Dana W. Olijhoek, Mette Olaf Nielsen, Natalja P. Nørskov

**Affiliations:** Department of Animal Science, Faculty of Technical Sciences, Aarhus University, Blichers Allé 20, 830 Tjele, Denmark; 201808215@post.au.dk (R.H.J.); maro@anis.au.dk (M.R.); mitho@anis.au.dk (M.T.); dana.olijhoek@anis.au.dk (D.W.O.); mon@anis.au.dk (M.O.N.)

**Keywords:** bioactive compounds, hemp, metabolomics, fractionation, methane mitigation

## Abstract

This study systematically evaluates the presence of methane mitigating metabolites in two hemp (*Cannabis sativa* L.) varieties, *Futura 75* and *Finola*. Hemp metabolites were extracted with methanol and fractionated using Solid Phase Extraction (SPE). Extracts, fractions, and the remaining pulp were screened for their methane mitigating potential using an in vitro model of rumen fermentation. The bioactive metabolites were identified with Liquid Chromatography-Mass Spectrometry (LC-MS). When incubated with a standard feed (maize silage), the extract of *Futura 75* significantly reduced methane production compared to that of control (without added extract) and without negative effects on feed degradability and volatile fatty acid patterns. The compounds responsible for the methane mitigating effect were assigned to flavonoid glycosides. However, none of the fractions of *Futura 75* or the pulp exhibited similar effect on methane emission. Butyric acid concentration in the fermentation inoculum was significantly increased, which could indicate why methane production was higher, when incubated with the fractions and the pulp. The extract of *Finola* did not show a similar significant effect, however, there was a numerical tendency towards lower methane production. The difference in methane mitigating properties between *Cannabis sativa* L. *Futura 75* and *Finola*, could be related to the content of bioactive flavonoids.

## 1. Introduction

Hemp (*Cannabis sativa* L.) has been identified as a bio-economic crop with multiple applicabilities and with low environmental footprint [1,2]. Organic hemp cultivation is increasing in Denmark and in the whole of Europe, and hemp-derived products already found their applications in medicine, construction, textile, cosmetics, as well as food industries and as feed supplements [1]. Recent interest in hemp is associated with several important environmental topics related to its cultivation. Hemp can be grown annually and has positive effects on the environment due to carbon sequestration, greenhouse gas (GHG)-emissions from soil, and crop rotation schemes in areas suitable for cash crop production [3,4]. In a report from the Danish Centre for Food and Agriculture (DCA), the climate foot print of hemp cultivation in Poland and the Netherlands was estimated to 1.69 and 1.56 Mg CO_2eq_ per hectare per year, respectively, which is lower than for winter wheat (3.10 and 1.67 Mg CO_2eq_ per hectare per year, respectively) [3]. Due to quick germination and growth, hemp outperforms weeds, which has a positive impact on the environmental footprint due to reduced need for fertilizers and pesticides [3]. However, the cultivation of hemp is strictly regulated by European authorities, and only hemp varieties with low contents of tetrahydrocannabinol (THC; <0.2%) are allowed to be cropped [5].

For hemp to be an economically valuable crop for farmers, it is important that all parts of the plant can be utilized. The fibers of hemp are used in construction and textile industries, whereas seeds are used for cosmetics and medicine [1]. However, there are several parts of the plant that have no industrial application yet, referring to leaves and the upper third part of the plant containing also stalks. Those parts of the plant are potentially digestible for ruminant animals but are often poorly digestible for monogastrics. Increasing the use of hemp biomass as feed additive could potentially lower the carbon footprint of dairy production systems, provided that hemp contains metabolites with methane mitigating potential. Ruminant animals are characterized by having large forestomachs, of which the rumen is the largest and hosts a multitude of bacteria capable of fermenting carbohydrates, including fibers that are indigestible for monogastric animals. During fermentation, CO_2_ and H_2_ are formed and converted into methane by methanogenic archaea. Methane is a GHG 28 times as potent as CO_2_ [6]. This is a challenge for the dairy and beef sectors in Denmark as well as globally, and effective ways to reduce enteric methane need to be found to mitigate global warming and climate change. 

For the last decade, untargeted metabolomics applying high-resolution mass spectrometry was successfully used to detect and identify bioactive compounds in many terrestrial plants, including hemp [7,8,9,10,11]. In the study by Delgado–Povedano et al., 2020 [10], untargeted metabolomics, Gas and Liquid Chromatography coupled to high resolution mass spectrometry time-of-flight (GC-TOF-MS and LC-TOF-MS) were applied to characterize the extracts of *Cannabis sativa* L. in 17 cultivars. Different chromatographic techniques were applied to increase metabolite coverage depending on their polarity. In total, 180 metabolites were identified with both chromatographic systems, 46 metabolites using LC-MS, and 134 metabolites using GC-MS [10]. Many terpenoids, lipids, cannabinoids, flavonoids, and fewer benzenoids, hydrocarbons, and amino acids were identified [10]. In another study, a comprehensive analysis of bioactive compounds in six fiber-type hemp inflorescences was performed by various analytical platforms, such as High Pressure Liquid Chromatography coupled to an Ultra Violet detector (HPLC-UV) and GC coupled to a Flame Ionization Detector (GC-FID) combined with MS-based platforms [7]. Moreover, the authors used different extraction solvents, such as acetone, ethanol, and ethyl acetate, for extraction of metabolites [7]. In the study by Delgado–Povedano et al., 2020 [10], metabolite classes such as cannabinoids, flavonoids, and terpenoids dominated the spectra. In the study of Jin et al., 2020 [11], the comprehensive analyses of secondary metabolites were performed on hemp plant parts; inflorescences, leaves, stem barks and roots. Inflorescences and leaves were characterized by cannabinoids, flavonoids and terpenoids, whereas stem barks and roots were mainly characterized by sterols and triterpenoids [11]. The highest concentrations of cannabinoids were found in inflorescences, whereas leaves contained the highest concentrations of flavonoids, such as orientin, vitexin, isovitexin, quercetin, luteolin, kaempferol, and apigenin [11]. These approaches all show that hemp produces a plethora of secondary metabolites with a diversity of known and unknown bioactivities. Cannabinoids for example have medical applications, whereas terpenoids and flavonoids were shown to have antimicrobial, anti-inflammatory, and antioxidative properties [12]. However, identification of compounds with particular bioactive properties is a difficult task, requiring extensive extraction and fractionation efforts. In the study by Lee et al., 2019 [8], untargeted metabolomics in combination with C_18_ Solid Phase Extraction (SPE) and preparative HPLC were successfully used to fractionate and identify antioxidative compounds in *Betulaceae* family plants. In another study, extraction and further C_18_ SPE fractionation were used to yield seven fractions of *Labisia pumila* [13]. A combination of C_18_ SPE, flash chromatography and HPLC was used to fractionate and identify bioactive compounds with anticholinesterase activity from *Phyllanthus muellarianus* [14]. One of the highly bioactive compounds in hemp, THC, was extracted and purified using superficial fluid extraction and SPE [15]. 

To our knowledge, no previous studies have been conducted to systematically identify and subsequently validate potential methane mitigating properties of hemp. The purpose of this study was: (1) to extract and systematically fractionate two industrially used hemp varieties, i.e., *Futura 75* and *Finola* (constituting the upper third part of the plant including leaves and stalks); (2) to assess the methane mitigating effect of the extracts, fractions, and the remaining pulp in an in vitro screening system simulating rumen fermentation; and (3) perform identification of potential methane mitigating compounds using LC-TOF-MS. We hypothesized that the two hemp varieties and derived extracts, fractions, and pulp differ in methane mitigating properties in vitro, which relates to the composition of bioactive compounds. 

## 2. Results and Discussion

### 2.1. Metabolic Profiling of Extracts and Fractions of the Hemp Varieties Futura 75 and Finola

Hemp was shown to contain thousands of metabolites with different bioactivities. The extraction and fractionation of hemp bioactive compounds are typically associated with cannabinoids and especially THC for pharmacological purposes. However, because hemp contains thousands of secondary metabolites, the methane mitigating potential of hemp was explored. 

**Figure 1:**
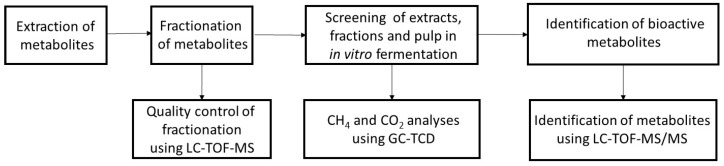
Schematic representation of the experimental design.

The extraction of hemp metabolites was performed with 75% methanol (MeOH) and the extract was further fractionated on C_18_ Solid Phase Extraction (SPE) columns with solvent of increasing lipophilicity at 25, 50 and 75% acetonitrile (ACN) in water. MeOH was used before to extract metabolites from hemp, such as flavonoid and cannabinoids [16,17]. Because MeOH extracts contain a high number of different metabolites with varying polarities and bioactivities, a systematic fractionation of the extracts was performed. This would thereby separate water-soluble from lipophilic compounds and minimize the number of metabolites per fraction for screening in an in vitro fermentation system for methane reduction and further in the identification of potential methane mitigating metabolites by LC-TOF-MS/MS. To compare the efficacy of fractionation, we also included unfractionated extracts and the remaining pulp in the in vitro screening. The schematic representation of the experimental design is shown in Figure 1. Detailed explanations of the extraction and fractionation procedures are described in the Material and Methods section. 

**Figure 2:**
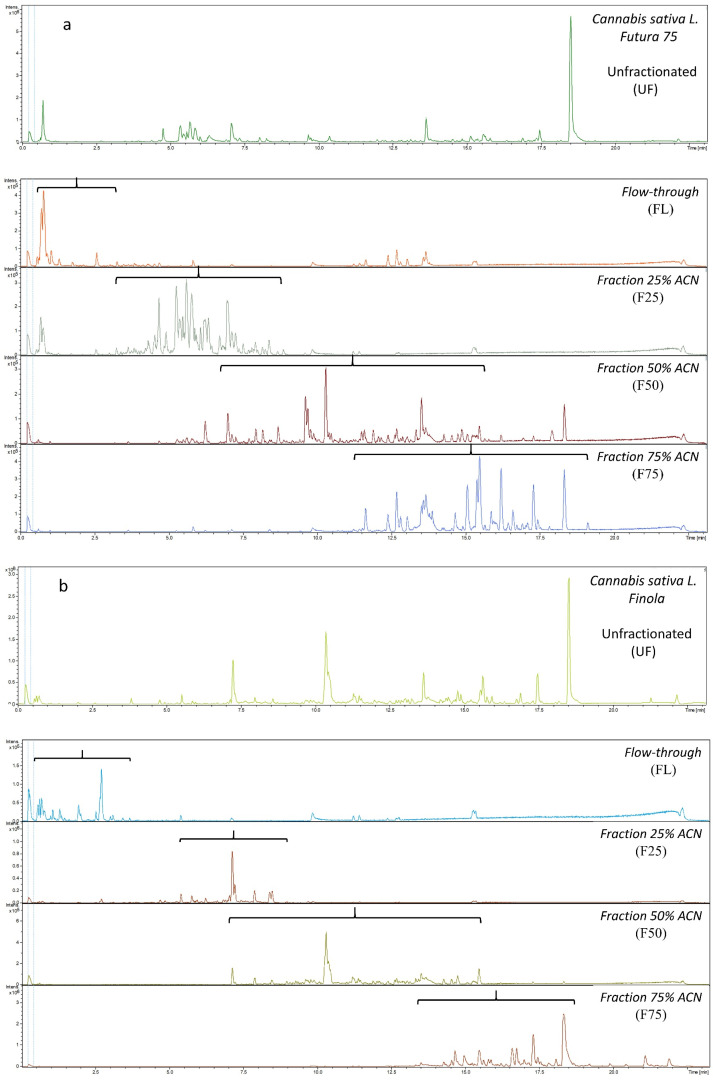
Metabolic profiles using LC-TOF-MS of *Cannabis sativa* L. *Futura 75* (**a**) and *Cannabis sativa* L. *Finola* (**b**), unfractionated, flow-through, and fractions 25%, 50%, and 75% acetonitrile (ACN) after evaporation and reconstitution in pure water.

Metabolic profiling using LC-TOF-MS was performed to qualitatively confirm the efficiency of extraction and fractionation of hemp metabolites according to their polarity. Figure 2 a and b show an unfractionated (UF) and four fractions of *Futura 75* and *Finola*, respectively, after evaporation and reconstitution in pure water. The chromatograms of LC-TOF-MS verified that the desired fractionation was achieved, when comparing the metabolic profiles of UF with the SPE fractionation. Metabolites were separated based on their hydrophobic properties into four fractions, though with some overlap between the fractions. The fractions flow-through (FL), 25% ACN (F25), and 75% ACN (F75) showed less overlap compared to fraction 50% ACN (F50), which had a broader metabolic profile. FL was characterized by elution of metabolites from approx. 0–3.5 min, F25 from approx. 3.5–9 min, F50 from approx. 6–16 min, and F75 from approx. 11.5–19 min. 

Reverse-phase chromatography is based on the principle of hydrophobic interactions, in which a hydrophobic stationary phase like C_18_ adheres molecules with different polarities carried by the polar mobile phase. When the polarity of the mobile phase decreases with increasing organic solvent, molecules elute at different time along the gradient, resulting in chromatographic separation [18]. Reverse-phase chromatography results in water soluble metabolites to elute first, followed by more lipophilic metabolites along the gradient of ACN. Generally, FL was therefore mainly characterized by the hydrophilic metabolites and F75 by lipophilic metabolites. The metabolic profiles in the four fractions from the two hemp varieties differed greatly. Cannabis plants can exhibit wide variations in the profiles of metabolites depending on the cultivar, growing conditions, and season [10]. The FL of *Futura 75* was defined by the elution of highly water-soluble compounds at the beginning of the chromatogram at 0.5 min, whereas the same fraction of *Finola* contained metabolites of higher hydrophobicity and intensity, eluting slightly later at 2.5 min. F25 of *Futura 75* contained a higher number of metabolites with high intensities compared to that of *Finola.* Similarly, F50 and F75 diverged in their metabolic profiles between these two varieties, however, both varieties contained high peaks of THC in F75 identified previously [16]. 

### 2.2. Screening for Methane Mitigation Potential and Identification of the Bioactive Compounds in Hemp Varieties Futura 75 and Finola

An in vitro system simulating rumen fermentation was used to screen for methane mitigating properties. In this system, a standard feed (0.5 g of maize silage) was incubated under anaerobic conditions in 90 mL inoculum composed of buffered rumen fluid without (control) or with addition to the inoculum of UF, FL, fractions and pulp derived from the two hemp varieties. Similar amounts of hemp biomass (0.5 g) were used for extraction and fractionation and equal volumes of reconstitution (6 mL).

Table 1 shows the effects of adding hemp extracts, fractions, or pulp to the inoculum on methane production, total gas production (TGP), volatile fatty acid (VFA) concentrations in the incubation media and degradability of organic matter (OM) in the maize silage after 48 h of in vitro fermentation. When UF of *Futura 75* was added to the incubation media, production of methane per gram sample OM, was significantly reduced compared to the control incubations with only maize silage. A decline in methane production was not observed with addition of FL, pulp, or any of the fractions. Addition of UF of *Finola* only caused a numerical reduction in the production of methane compared to that of control. UF from *Finola* did, however, induce a significantly lower methane production as compared to pulp. None of the fractions or FL of *Finola* inhibited production of methane compared to that of control. 

A similar pattern was observed for TGP, except that TGP for pulp of *Finola* was significantly higher compared to that of control. None of the tested additives influenced the degradability of OM in the maize silage nor the concentration in the incubation medium of total VFAs derived from fermentation of the feed. However, concentration of butyric acid was significantly increased by the addition of all three fractions, FL, and the pulp, but not by the addition of UFs derived from *Futura 75*, compared to that of the control. Significantly higher production of butyric acid was also observed upon addition of pulp or F50 from *Finola*, but not for UF, FL, F25, and F75.

The results of the in vitro screening showed that extracted metabolites from *Futura 75* have the potential to inhibit methane production in vitro without changing the total VFA production or degradability in the rumen of a standard feed, which is important in relation to the production economy, if inclusion of methane mitigating feed additives to diets of ruminants should be considered in the future. Although the fractions of *Futura 75* did not inhibit methane production, they affected the proportion of butyric acid formation relative to other VFAs produced during fermentation of the feed. 

**Figure 3:**
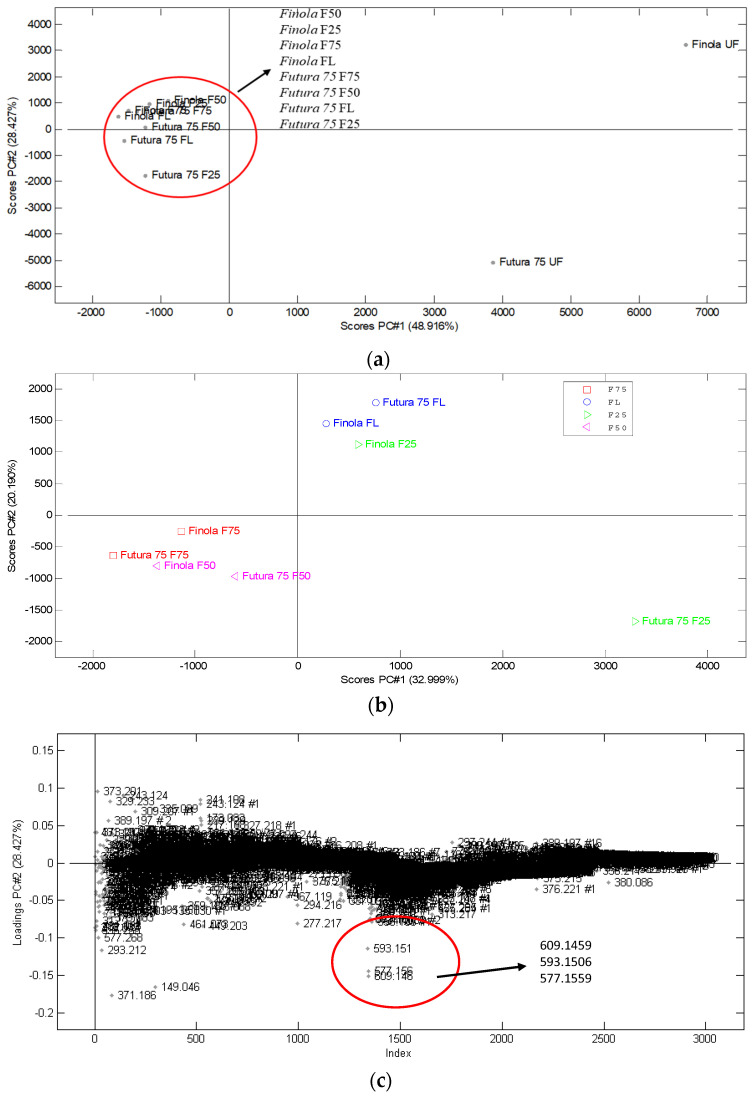

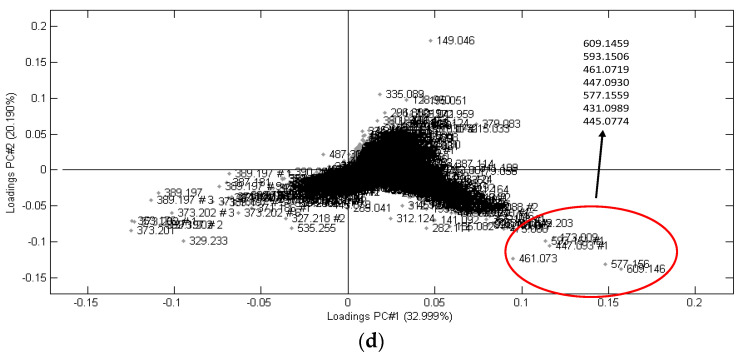
Principal Component Analyses (PCA) scores plots (**a**,**b**) and their corresponding loadings plots (**c**,**d**). Scores plot (**a**) shows discrimination between unfractionated extracts of *Cannabis sativa* L. *Futura 75* and *Cannabis sativa* L. *Finola* (UF) and their corresponding fractions; flow-through (FL), 25% ACN (F25), 50% ACN (F50), and 75% ACN (F75). Scores plot (**b**) shows grouping of fractions FL, F50, and F75 and discrimination between F25 of *Cannabis sativa* L. *Futura 75* and *Cannabis sativa* L. *Finola*. Loading plots (**c**,**d**) show m/z of metabolites responsible for discrimination.

To identify metabolites responsible for these effects, a Principal Component Analyses (PCA) combined with MS/MS spectra for metabolites identification was performed. The PCA´s in Figure 3a–d show the scores and loadings plots of *Futura 75* and *Finola* and their fractions. Metabolites responsible for discrimination were identified to belong to the class of phenolic compounds known as flavonoids glycosides. The following flavonoids glycosides with varying length of sugar moities were tentatively identified (Table 2): kaempferol, vitexin, orientin, deosmetin, genistein, and daidzein; their corresponding MS/MS spectra are included in the Supporting Information. These secondary metabolites were mainly present in *Futura 75* and separated by PC2, Figure 3a,c. The PCA analyses also showed that FL, F50 and F75 of *Futura 75* and *Finola* contained predominantly similar metabolites, grouping closely together on the scores plots and oppositely to F25 (Figure 3b). The main discrimination between F25 of *Futura 75* and *Finola* was also attributed to flavonoids glycosides, shown on the corresponding loadings plot, Figure 3d. The highest discrimination on PC1 was achieved between unfractionated extracts and the fractions (Figure 3a), which was expected, because fractions do not contain the same number of metabolites compared to that of UF. PC1 discriminated also between FL, F25, and F50, with F75 reflecting differences in the contents of hydrophilic and lipophilic compounds in the fractions (Figure 3b).

The tentative identification of flavonoids glycosides was based on their fragmentation patterns of MS/MS spectra compared to the literature and the Human Metabolome Database (HMDB). The characteristic fragmentation of flavonoids glycosides using neutral loss is well described in the literature [20]. The neutral loss of (M-H-90)^−^ and (M-H-120)^−^ was previously described by Kazuno et al., 2005 [20] for C-Glycosides. The neutral loss of (M-H-176)^−^ is typical for glucuronic acid conjugates and the loss of (M-H-44)^−^ corresponds to the loss of CO_2_ [22]. However, flavonoids can be bound to several sugar moieties, which increase their molecular weight, and can be characterized during collision induced dissociation as a loss of high molecular weight fragments visible in the MS/MS spectra (Supporting Information).

The extraction of plant secondary metabolites and further screening for bioactivity is a widely used technique, which was previously performed for a variety of plants, but to the best of our knowledge never for hemp. In the study by Bodas et al., 2008 [23], 450 plant species were screened in vitro for effects on methane production, and they reported a 15% methane depression for six plants; *Carduus pycnocephatus*, *Populus tremula*, *Prunus avium*, *Quercus robus*, *Rheum nobile*, and *Salix caprea* [24]. No adverse effects on feed degradability, total gas and VFA production were observed [23]. Kamra et al., 2008 [25] screened 93 plant extracts for their potential to inhibit in vitro methane production and ciliate protozoa. The results demonstrated that 20 out of 93 plant extracts lowered methane by more than 25% and decreased methanogen numbers [24,25,26]. However, the identification of bioactive metabolites is not revealed when screening for bioactivity. Further exploration and fractionation are necessary to identify compounds of interest. 

In this study, extracts of hemp varieties *Futura 75* and *Finola*, their fractions and pulp were screened for methane mitigation potential. The extract of *Futura 75* showed significant methane mitigating potential without any detrimental effects on degradability of the basal feed or on VFA concentation profiles (rumen fermentation patterns), and metabolites responsible for the methane mitigating effects were identified in the current study to belong to the class of phenolic compounds known as flavonoids glycosides: kaempferol, luteolin, apigenin, vitexin, and dismetin. Flavonoids were previously shown to decrease methane production in vitro [24,27]. In a screening study with 13 plants, flavonoid-rich extracts of *Equisetum arvense* and *Salvia officinalis* were shown to decrease methanogenesis by 8 to 14% [28]. In the study of Oskoueian et al., 2013 [29], the inclusion of flavone, myricetin, naringin, rutin, quercetin, and kaempferol decreased methane production at concentrations of 4.5% of the substrate (dry matter (DM) basis). Their potential ranked as follows: myricetin ≥ kaempferol ≥ flavon ≥ quercetin ≥ naringin ≥ rutin ≥ catechin [29]. In another study, the evidence for a hydrogen-sink mechanism of catechin was demonstrated [30]. Extracts rich in mixed flavonoids and pure flavonoid compounds added in concentration of 0.2 g/kg DM decreased relative abundances of hydrogenotropic methanogens [31]. 

Although fractionation of the extract of *Futura 75* was achieved, none of the fractions exhibited similar potential as the unfractionated extract. The largest difference in PCA analyses between *Futura 75* and *Finola* could be attributed to the presence of the bioactive flavonoids in *Futura 75*, which were also present in F25. Therefore, the lack of effect of F25 could be due to too low concentrations of flavonoids and/or synergetic effects with other metabolites. Another explanation can be reflected in the production of butyric acid, which was significantly higher, when the fractions were added to the incubation media, but not upon addition of the UF. Because phenolic compounds are bound in the cell wall matrix by sugar molecules or conjugated with glucuronic acid, the sugar becomes more bioaccessible for fermentation, when extracted and fractionated compared to the unfractionated extract [32,33]. *Beta*-glucuronidase is a common enzyme, which is involved in the deconjugation of phenolic compounds by microorganisms [34], and released sugars and sugar acids can be used for fermentation resulting in increased butyric acid formation. However, the added amount of sugar via this route to the incubation medium may perhaps have been too low to explain the quantitative changes in butyric acid concentrations in the medium by the end of the 48h fermentation period. However, certain microorganisms can exploit sugar acids as nutritional niche for their growth, as it was suggested for catabolism of gluconate in *E. coli* [35], thereby creating differences in the fermentation patterns, but this requires further investigation. 

The higher bioactivity of *Futura 75* compared to *Finola* may not only be related to differences between varieties, but could also be related to differences in the production of secondary metabolites due to harvest year and period [11,36,37]. Previously, the content of secondary metabolites was shown to vary between different cultivars. However, the largest variation can be ascribed to the year of harvest, which relates to the growing conditions and harvest time (the phenological stage of the plant). In the study by Irakli et al., 2019 [36], TPC of seeds of *Futura 75* and *Finola* were compared during two years, 2017 and 2018. For the year 2017, no significant difference was observed between the two cultivars, whereas in year 2018, a significant difference in TPC was measured [36]. In the study of Andre et al., 2020 [37], the main influence on the TPC and the individual flavonoids of inflorescence was observed for harvest period (early or late summer) and the phenological stage of the plant. At the end of summer or beginning of spring, the TPC was nearly half of the concentration in July and was also influenced by the flowering stage of the plant [37]. Jin et al., 2020 [11] also reported that the concentration of individual flavonoids and TPC decreased as the plant aged. In addition, the concentration of flavonoids varied between different parts of the plants, with the highest concentration found in leaves and inflorescences [11]. Since both cultivars in this study were harvested by the same technique to collect the upper third part of the stem (containing stalks and leaves), we suggest that a major difference in flavonoid content may be related to differences in harvest time, as *Futura 75* was harvest in early summer, whereas *Finola* was harvested in late summer. 

## 3. Materials and Methods

### 3.1. Chemicals

Methanol was HPLC grade from Sigma (Merck KGaA, Darmstadt, Germany). Acetonitrile (ACN), isopropanol (VWR, West Chester, PA, USA) and formic acid were LC-MS grade from Fluka (Merck KGaA, Darmstadt, Germany). Lithium formate monohydrate 98% was purchased from Sigma (Merck KGaA, Darmstadt, Germany). Methane (769126-1L) Messer^®^ CANGas 99.995% and Carbon dioxide (769002-1L) Messer^®^ CANGas 99.995% were from Sigma-Aldrich (Darmstadt, Germany).

### 3.2. Sample Information

**Figure 4:**
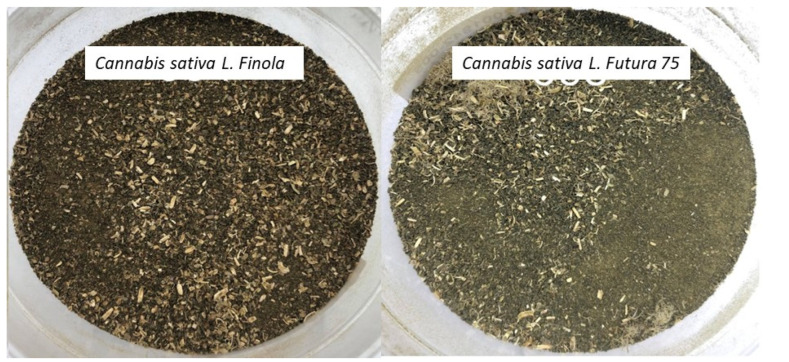
Milled hemp samples of *Cannabis sativa* L. *Finola* and *Cannabis sativa* L. *Futura 75*.

*Cannabis sativa* L. *Futura 75* and *Cannabis sativa* L. *Finola* were harvested in Denmark on the 24 June 2018 and 31 August 2019, respectively. The upper third part of the stem containing stalks and leaves were harvested and dried for five days, while the seeds from the plants were threshed. The dry hemp was industrially milled before it was milled a second time at Aarhus University to pass through a 1-mm screen. The samples were homogenized using an Ultra Centrifugal Mill ZM 200 (Retsch, Haan, Germany), Figure 4.

### 3.3. Extraction and Fractionation Protocol

**Figure 5:**
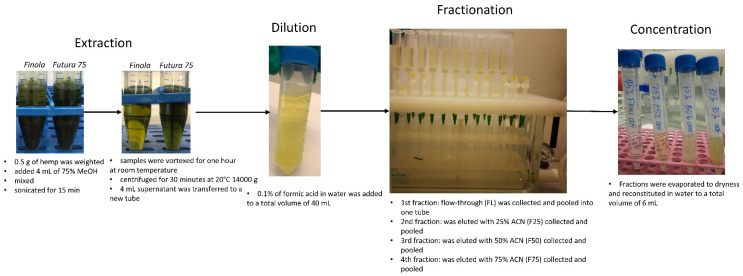
Protocol of systematic extraction and fractionation of *Cannabis sativa* L. *Futura 75* and *Cannabis sativa* L. *Finola*.

Milled hemp samples (0.5 g) were dissolved in 4 mL 75% MeOH, mixed and sonicated for 15 min and further vortexed for 1 h at room temperature to extract metabolites, Figure 5. To separate the pellet from the supernatant containing extracted metabolites, the samples were centrifuged for 30 min at 20 °C 14,000 g and the supernatant was transferred to a new tube. The pellet was allowed to dry for three hours and was further used as the pulp fraction. The supernatant was either directly evaporated to dryness for 18–26 h using a ScanSpeed/SCANVAC vacuum centrifuge from (LH Laboratorie Service A/S, Denmark) and reconstituted in 6 mL of water or used for fractionation. A small aliquot of unfractionated extract was used for qualitative control using LC-TOF-MS. Fractionation: 4 mL of extract diluted 10× with 0.1% formic acid in water to a total volume of 40 mL to increase the affinity of extracted metabolites towards C_18_ SPE columns. Fractionation was performed on C_18_-E SPE columns (2 g, 6 mL) attached to a vacuum manifold from Phenomenex (Torrance, CA, USA). The columns were activated with 4 mL of 100% ACN and 2 × 4 mL of water. Four mL out of the 40 mL solution were added to ten separate C_18_ SPE columns. The flow-through was collected into ten tubes. The metabolites that had the affinity towards the C_18_ SPE columns were further collected by elution with 2 mL 25% ACN to each SPE column, followed by 50% and finally 75% ACN. In total, four fractions were hence collected from each hemp variety. After fractionation, 10 tubes from each fractionation were pooled into one tube and mixed. An aliquot from each pooled tube (representing one fraction) was transferred to a vial for qualitative LC-TOF-MS analyzes, to verify the metabolic profiling and fractionation of metabolites. The pooled samples were further evaporated to dryness for 18–26 h in 5 mL aliquots using a vacuum centrifuge. After evaporation, the samples were reconstituted in 1 mL of water per glass and shaken for 1 h. The total volume of each fraction after evaporation and reconstitution was 6 mL, Figure 5. After evaporation and re-constitution, an aliquot of each fraction was taken for quality control of metabolic profiling and fractionation by LC-TOF-MS. 

### 3.4. Metabolic Profiling Using LC-TOF-MS

Chromatographic separation was performed on Ultra-Performance Liquid Chromatography (UPLC) Ultimate 3000 (Dionex, Sunnyvale, CA, USA), which was connected to an impact HD quadrupole Time-of-Flight (QTOF) mass spectrometer from Bruker Daltonics (Bremen, Germany) operated in full scan mode from 50 to 1000 m/z at a sampling rate of 1 Hz. LC-TOF-MS was performed according to a previously developed protocol [16]. Briefly, the UPLC was equipped with the precolumn filter Van Guard Precolumn, 2.1 × 5 mm and column HSS T3 C_18_ UPLC column 1.8 um, 100 × 2.1 mm from Waters (Milford, MA, USA). The column oven was set to 30 ˚C, the temperature of the autosampler was 5 °C, the injection volume was 8 µL and the flow was set to 400 µL/min. The solvent system consisted of solvent A (0.1% formic acid in water) and solvent B (0.1% of formic acid in ACN). The gradient started at 5% acetonitrile and continued to 80% during 23 min with post- and pre-equilibration of 2 min. The measurements were performed in negative ionization mode using electro spray ionization (ESI). Mass shifts were corrected by use of external equilibration with lithium formate clusters at a concentration of 5 mM dissolved in a solvent of water-isopropanol-formic acid. Capillary and end plate offset were set to −4500 and −500 V respectively. The dry gas flow was set to 8 L/min, ion source temperature to 200 °C and nebulizer pressure to 1.8 bar. Collision energy during MS scan was set to 6 eV. For MS/MS analyzes, argon gas was used as a collision gas and auto-MS/MS was performed with collision energies between 10 and 40 eV. Samples were subjected to MS scan for compound detection and auto-MS/MS scan for compound identification. Identification of compounds was performed by comparing accurate mass and MS/MS spectra with published literature and the online Human Metabolome Database (HMDB).

### 3.5. Working Solutions for In Vitro Fermentation System

The working solutions for the in vitro fermentation system included redox indicator, reducing agent, buffer, and macro- and micro-mineral solutions. The solutions were prepared as described by Menke and Steingass 1988 [38] and kept under anaerobic conditions by continuously flushing with N_2_ until added to incubation bottles. The ratio between buffer solution and rumen fluid was 2:1. 

### 3.6. Rumen Fluid

Rumen fluid was collected from three rumen cannulated non-pregnant dry Holstein cows (approx. 695 kg) housed at the experimental facility at Aarhus University, Foulum, Denmark. The handling and care of the cows complied with the guidelines set out by the Danish Ministry of Environment and Food (2020) (Act No. 2028, 2020) with respect to animal experimentation and care of animals under studies. The cows were fed at maintenance level on a standard diet consisting of straw, hay, and a concentrate mixture, as described by Brask et al., 2013 [39]. The fluid and particles were collected in preheated thermo bottles half an hour before morning feeding and transported to the laboratory within 30 min of sampling. At the laboratory, the rumen fluid was filtered through two layers of moist cheesecloth, and pH was measured.

### 3.7. In Vitro Fermentation System

To determine the effect of UF, F25, F50, F75, and pulp of *Futura 75* and *Finola* on methane formation during in vitro rumen fermentation, 0.5 g of maize silage, which served as a standard feed, was weighed into Duran^®^ bottles (capacity: 132 ± 1.1 mL) and 1 mL of UF or fraction or 0.1 g of pulp were subsequently added to the maize silage. Bottles containing only maize silage without any additives served as controls.

Ninety milliliters of buffered rumen fluid inoculum were then added to each bottle. The headspace was flushed with N_2_ and then an ANKOM pressure sensor module (AnkomTechnology, Macedon, NY, USA) was fitted on top of the bottle. Bottles with samples were incubated at 39 °C for 48 h in a controlled incubator shaker (New Brunswick^TM^ Excella^®^ E25R, Eppendorf, Hamburg, Germany) with the oscillation set at 50 rpm. Pressure changes in the headspace of the bottles during the incubations were measured continuously as a difference with respect to the concurrently measured atmospheric pressure. Recordings were obtained at intervals of 10 minutes via radio frequency to a computer. Accumulated gas in the headspace was automatically released (250 milliseconds vent opening) into a gas-tight FlexFoil bag (1 L; SKC Ltf, Dorset, United Kingdom) attached to the unit, when the pressure inside the bottle reached 0.75 psi above ambient pressure. Each sample type (extract, fraction, or pulp) was analyzed as triplicates in two separate runs. Each run also included three replicates of pure maize silage without any additives as a control sample, and three replicates of only rumen inoculum, which served as blank samples. Beforehand, the maize silage had been freeze-dried and milled through a 0.5 cm sieve on a centrifugal mill (Ultra Centrifugal Mill ZM 200, Verder Scientific, Hann, Germany). 

After 48 h of incubation, 10 mL of gas was extracted from each gasbag using a polypropylene syringe (BD Plastipak, Madrid, Spain) and transferred into evacuated GC-vials (Labco Limited, Ceredigion, United Kingdom) for later CH_4_ and CO_2_ analyses. The inoculum with undegraded feed residue was filtered through F57 fiber bags (ANKOM Technology, Macedon, NY, USA) for collection of undegraded feed residues, and a sample of the flow through was collected for VFA analysis. 

### 3.8. CH_4_, CO_2_ and VFA Analyses Using GC-TCD

The GC-TCD analyzes of CH_4_ and CO_2_ were performed using a Trace 1310 GC with TCD detector and a TriPlus Headspace autosampler (Thermo Fisher Scientific, Waltham, MA, USA). The injection volume was 200 µL with an inlet temperature of 150 °C. The sample was injected in split mode, and CH_4_ and CO_2_ were separated on a HP-FFAP column, 30 m length, ID 0.53 mm and 1 µm film thickness (Agilent Thechnologies, Wilmington, NC, USA). The temperature program for the column was as follows: initial temperature, 50 °C (1.75 min hold), followed by the gradient to 150 °C at 20 °C/min and held for 1 min, the chromatographic separation was 8.75 min. The flow rate of the carrier gas (He) was 1.7 mL/min. The temperature of the TCD detector was 200 °C. Quantification was performed using standard curves for CH_4_ and CO_2_ with standard gasses in concentrations ranging from 100–200.000 ppm added to Chromilion 7.2.10. Exetainer^®^ 5.9 mL vials (Labco, Lampeter, UK). 

VFA analyses were performed as described by Kristensen et al., 2009 [40], using a Rt^®^-Q-BOND column, 30 m length, ID 0.25 mm and 8 µm film thickness (Restec, Bellefonte, PA, USA). 

### 3.9. Chemical Analyses

The contents of DM and OM in the standard feed (maize silage), undegraded feed residues and hemp pulp were determined in duplicate and used for calculation of DM and OM addition to bottles in the incubated samples and for calculation of the rumen degradability of DM and OM over 48 hours of incubation. DM content was determined by oven drying at 103 °C overnight, while the ash was determined by combustion at 525 °C for 6 h. Degradable DM (%) was calculated as: (total DM (feed +/− pulp) added to incubation bottles minus DM content in the undegraded feed residues after incubation)/(total DM added to incubation bottles) × 100. 

### 3.10. Calculations and Statistical Analyses

The cumulative gas pressure recorded during the 48 hour fermentation period was converted to mL gas produced at standard temperature and pressure (STP) (IUPAC, 2012) [41] assuming that the ideal gas law applies to the produced gasses (predominantly CO_2_, CH_4_ and H_2_). TGP and degradable DM response parameters were blank corrected before the statistical analyses. Methane (mL) production was calculated from TGP after 48 h and the CH_4_ concentration (%) in the collected gas. 

The statistical analyses were conducted in R 4.1.2 (R Core Team, 2020). Effects of the hemp extracts, fractions and pulp on the various response parameters were analyzed with the following linear mixed model: Y_tbe_ = μ + α_t_ + γ·s_be_ + A_e_ + ɛ_tbe_, where Y_tbe_ is the dependent response variable, μ is the overall mean, α is the fixed effect of treatment (t = 1 to 13), γ is the fixed regression parameter of starting time and s_be_ denotes the starting time of the b’th bottle of run e, A is the random effect of experimental run (e = 1 to 4), and ɛ_tbe_ is the random residual error, assumed to be independent with constant variance and normally distributed. The data were tested for normality of the residuals by evaluating the QQ-plots constructed in R and by using the Shapiro–Wilk test. Homogeneity of the variance was tested by evaluating plots of residuals and by using Bartlett’s test. Data are presented in tables as estimated marginal means (EMS) and standard error of means (SEM). Two-way analysis of variance (ANOVA) was used to compute the *p*-values for the fixed effects. Differences between EMS were evaluated using Tukey’s method for comparison. Statistical significance was declared when *p* ≤ 0.05 and statistical tendencies were declared when 0.05 < *p* ≤ 0.10.

### 3.11. Preprocessing of Metabolomics Data and Multivariate Data Analysis

The raw data files were converted to mzXML files using CompassXport (Bruker Daltonics, Bremen, Germany). The software MZmine 2 [42,43] was used to preprocess the data. Centroid data peaks of negative mode were detected using the ‘centroid mass detector’ with noise level of 2500. Chromatograms were built using ‘ADAP chromatogram builder’, deconvoluted using the ‘Wavelets (ADAP)’ algorithm and deisotoped using the ‘isotopic peaks grouper’. Chromatograms were aligned using the join aligner. For each option m/z tolerance of 0.01 m/z or 5 ppm and RT tolerance of 10 s (=0.25 min) were used. Gaps (missing peaks) were filled using the option ‘Same RT and m/z range gap filler’ with m/z tolerance of 0.001 m/z or 5 ppm. Last, duplicate peaks were filtered using filter mode ‘new average’ with m/z tolerance of 0.01 m/z or 5 ppm and RT tolerance of 0.25 min. Data were exported to Excel and further analyzed as PCA in LatentiX version 2.13 (LatentiX Aps, Gilleleje, Denmark) using Pareto scaling prior to analysis.

## 4. Conclusions

Our results, based on an in vitro model simulating rumen fermentation, indicate that flavonoids of *Cannabis sativa* L. variety *Futura 75* have the potential to inhibit enteric methane production without negative effects on degradability in the rumen of the standard diet or the rumen fermentation (VFA) patterns. Although a satisfactory fractionation of compounds from *Cannabis sativa* L. variety *Futura 75* was achieved, none of the derived fractions lowered methane production from the standard feed in vitro. The lack of effect of the fractions is hypothesized to be related either to the loss of synergetic effects among different metabolites due to fractionation or to a higher bioaccessibility of sugars and sugar acids derived from flavonoids glycosides, which possibly influenced fermentation patterns by rumen microorganisms. However, these hypotheses require further investigation. *Cannabis sativa* L. variety *Finola* did not exhibit a significant effect on methane production, however, there was a numerical tendency towards lower methane production. This difference between two varieties of hemp was attributed to differences in contents of flavonoids glycosides. 

## Figures and Tables

**Table 1:** Effects of addition of hemp extract (UF), flow-through (FL), fractions eluted with 25% acetonitrile (ACN) (F25), 50% ACN (F50), 75% ACN (F75), and pulp to a standard feed (0.5 g maize silage) on dry matter (DM) degradability, total gas production (TGP), methane (CH_4_) production per sample organic matter (OM), and volatile fatty acid (VFA) concentrations in the incubation media after 48 h incubation in an in vitro system simulating rumen fermentation. Two varieties *Cannabis sativa* L. *Futura 75* and *Cannabis sativa* L. *Finola* were used in this study.

	CH_4_/Sample OM (mL/g)	TGP (mL)	Degradable DM (%)	Total VFA (mmol/L)	Acetic Acid (% of Total VFA)	Propionic Acid (% of Total VFA)	Butyric Acid (% of Total VFA)	Total Other ^1^ VFAs (mmol/L)	Other VFAs (% of Total VFA)
Maize silage	61.6 ^ab^	79.7 ^b^	60.5 ^ab^	127	40.2	30.2	2.28 ^b^	3.19	2.52
*Futura 75* UF	41.2 ^c^	60.6 ^c^	58.6 ^ab^	113	46.3	26.0	3.19 ^ab^	3.67	3.31
*Futura 75* FL	58.9 ^abc^	81.5 ^abc^	64.3 ^ab^	110	47.0	25.1	3.49 ^a^	3.86	3.56
*Futura 75* F25	59.0 ^abc^	77.4 ^ab^	62.1 ^ab^	104	50.0	23.7	3.79 ^a^	3.98	3.82
*Futura 75* F50	58.3 ^abc^	80.4 ^ab^	66.4 ^a^	113	46.3	25.6	3.46 ^a^	4.03	3.64
*Futura 75* F75	57.9 ^abc^	76.3 ^ab^	65.3 ^ab^	109	49.3	24.6	3.66 ^a^	4.04	3.81
*Futura 75* Pulp	62.1 ^abc^	86.6 ^ab^	51.2 ^b^	114	47.6	25.4	3.68 ^a^	4.07	3.63
*Finola* UF	46.3 ^bc^	63.1 ^ab^	68.9 ^ab^	112	47.7	25.6	3.29 ^ab^	3.67	3.43
*Finola* FL	62.5 ^abc^	83.7 ^abc^	63.1 ^ab^	111	46.9	25.2	3.50 ^ab^	3.92	3.56
*Finola* F25	59.5 ^abc^	77.0 ^ab^	69.2 ^a^	120	44.7	27.1	3.40 ^ab^	4.03	3.38
*Finola* F50	59.7 ^abc^	79.8 ^ab^	63.2 ^ab^	107	50.3	24.1	3.81 ^a^	4.01	3.80
*Finola* F75	62.9 ^ab^	81.3 ^ab^	61.7 ^ab^	117	45.0	26.3	3.35 ^ab^	3.90	3.37
*Finola* Pulp	73.7 ^a^	97.2 ^a^	57.4 ^ab^	121	47.1	27.4	3.61 ^a^	4.30	3.62
SEM	4.38	4.74	6.11	11.3	5.37	2.45	0.463	0.347	0.579
*p* value	0.04	<0.001	0.03	0.50	0.58	0.17	0.03	0.12	0.18

^a–c^ Values within the same column with different superscripts are significantly different (*p* ≤ 0.05). ^1^ Valeric, isovaleric and caproic acids.

**Table 2:** Tentatively identified flavonoids glycosides in *Cannabis sativa* L. *Futura 75*.

RT ^1^ (min)	M-H^−^ (m/z) (Δppm)	Fragments (m/z)	Neutral Loss (Da)	AutoMS/MS (eV) ^2^	Tentatively Identified
5.35	609.1458 (0)	489.1042, 429.0823, 357.0611, 327.0510, 309.0409	120, 60	22–28	Kaempferol 7-sophoroside (HMDB)
5.46	593.1506 (1)	473.1092, 429.0813, 357.0619, 327.0509, 309.0399	120, 44, 72	22–27	Kaempferol-3-*O*-neohesperidoside [19]
5.55	447.0930 (1)	357.0618, 327.0511, 297.0405	90, 120	19–24	Orientin [20,21]
5.55	895.1917	447.0929, 357.0610, 327.0505	448, 90, 120	28–35	Orientin + sugar moiety
5.66	593.1506 (1)	473.1068, 413.0869, 293.0449	120, 60, 120	22–27	Kaempferol diglycoside (HMDB)
5.66	875.2248	593.1506, 473.1076, 413.0881, 293.0452	282, 120, 60, 120	28–34	Kaempferol diglycoside + sugar moiety
5.77	577.1559 (1)	457.1139, 413.0880, 311.0563, 293.0453	120, 44, 120	22–27	Daidzein diglycoside (HMDB)
6.01	431.0989 (1)	341.0672, 311.0561, 283.0611	90, 120	19–23	Vitexin [21]
6.01	863.2014	431.0989, 341.0654, 311.0557	432, 90, 120	27–34	Vitiexin + sugar moiety
6.30	461.0719 (2)	285.0400	176	19–24	Kaempferol glucuronide (HMDB)
6.30	923.1496	461.0726, 285.0403	462, 176	29–36	Kaempferol glucuronide + sugar moiety
7.05	445.0774 (1)	269.0451	176	19–24	Genistein glucuronide (HMDB)
7.05	668.1190	445.0768, 269.0453	223, 176	23–30	Genistein glucuronide + sugar moiety
7.05	891.1607	445.0769, 269.0450	446, 176	28–35	Genistein glucuronide + sugar moiety
7.32	475.0876 (2)	299.0556	176	19–24	Diosmetin glucuronide (HMDB)
7.32	951.1803	475.0876, 299.0553	476, 176	29–36	Diosmetin glucuronide + sugar moiety

^1^ Retention Time. ^2^ Collision energy.

## Data Availability

The data presented in this study are available on request from the corresponding author. The data are not publicly available due to security policy of the Aarhus University.

## Supplementary Materials

The following are available online at https://www.mdpi.com/article/10.3390/metabo12010077/s1, Supplementary material contain MS/MS spectra of identified flavonoids glycosides.
