# Supplementary material for: Untargeted Metabolomics Combined with Solid Phase Fractionation for Systematic Characterization of Bioactive Compounds in Hemp with Methane Mitigation Potential
Source: Metabolites. 2022 Jan 13;12(1):77. doi: 10.3390/metabo12010077 (PMC8779194; doi:10.3390/metabo12010077)

Suplemented material

Kaempferol 7-sophoroside

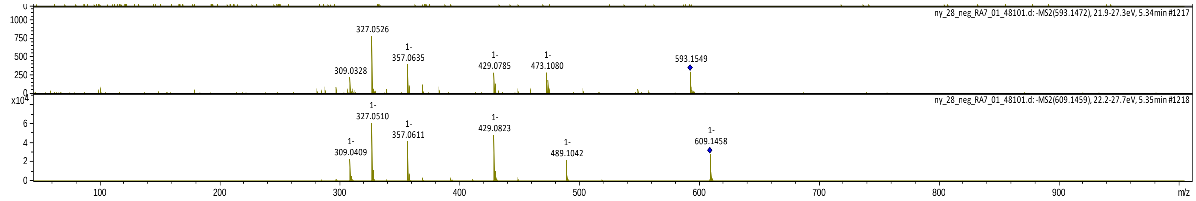

Kaempferol-3-*O*-neohesperidoside

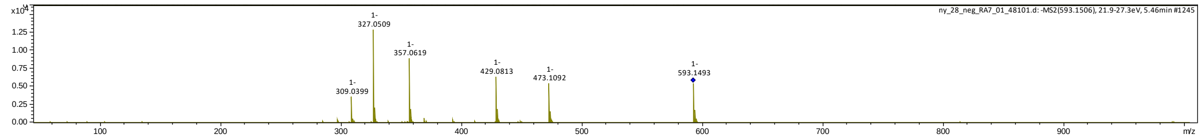

Orientin and Orientin + sugar moiety

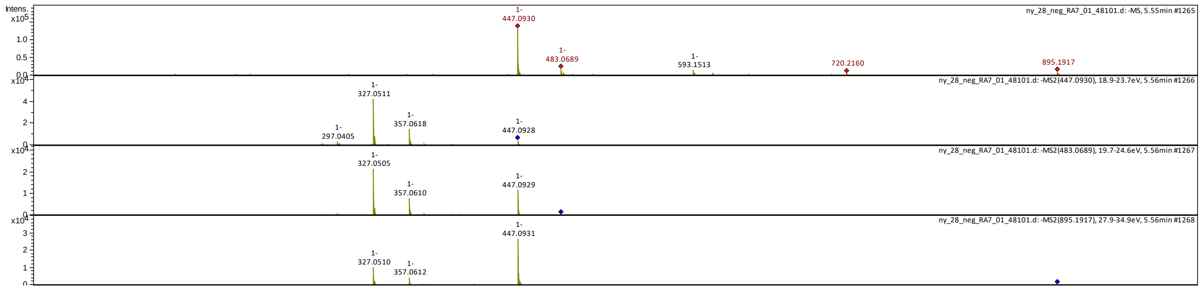

Kaempferol diglucoside and Kaempferol diglucoside + sugar moiety

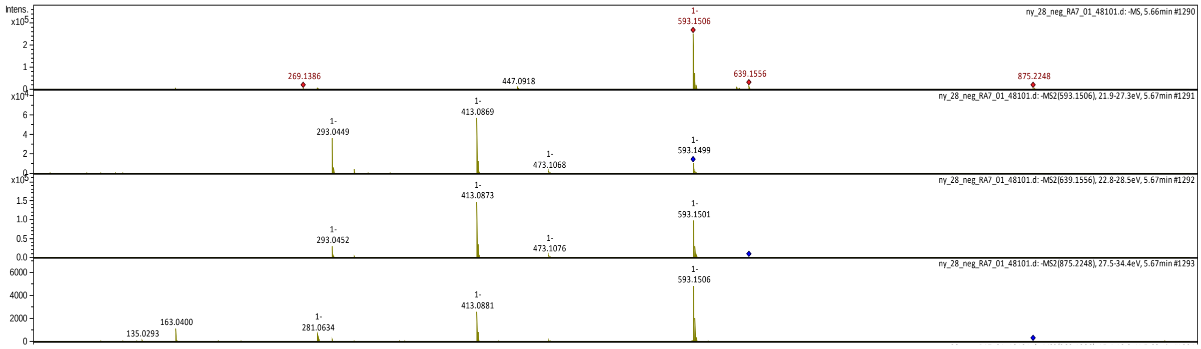

Daidzein diglucoside

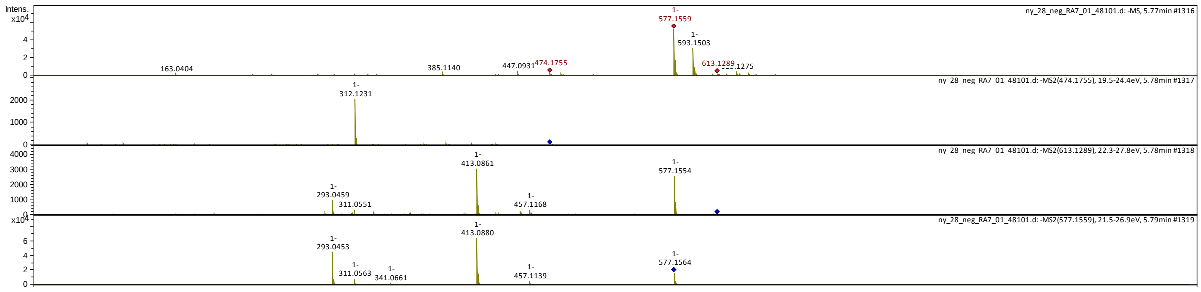

Vitexin and Vitiexin + sugar moiety

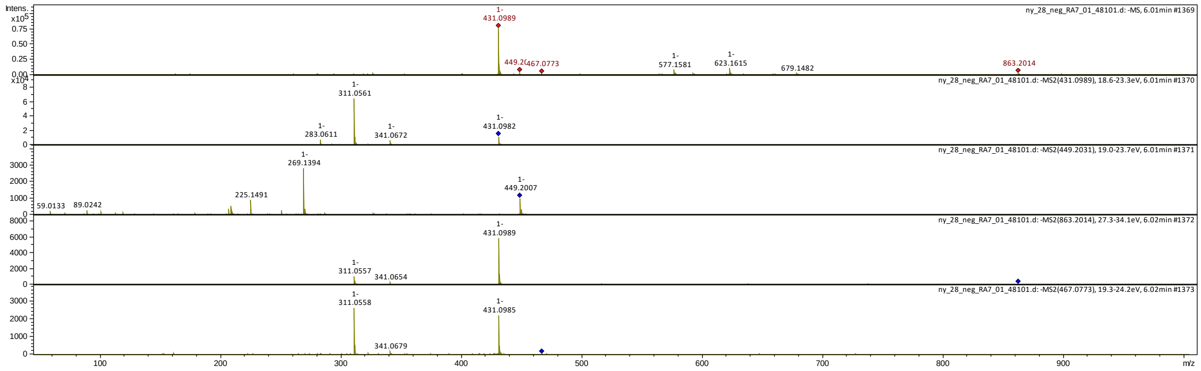

Kaempferol glucuronide and Kaempferol glucuronide + sugar moiety

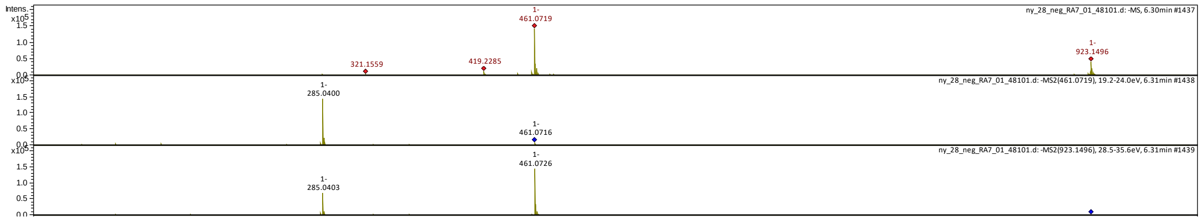

Genistein glucuronide and Genistein glucuronide + sugar moiety

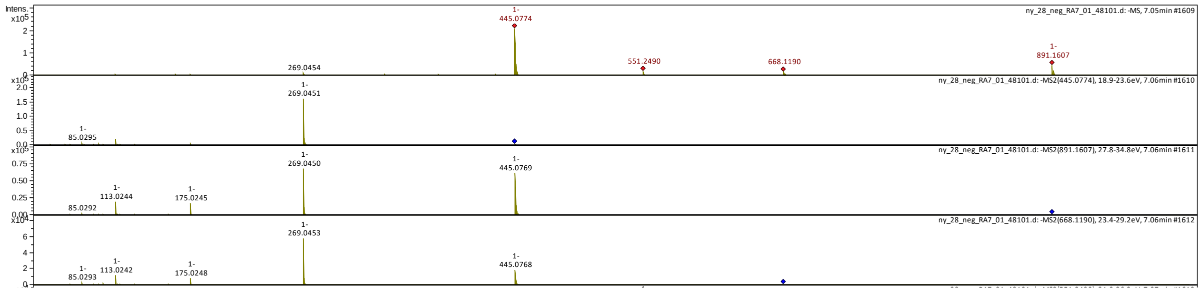

Diosmetin glucuronide and Diosmetin glucuronide + sugar moiety

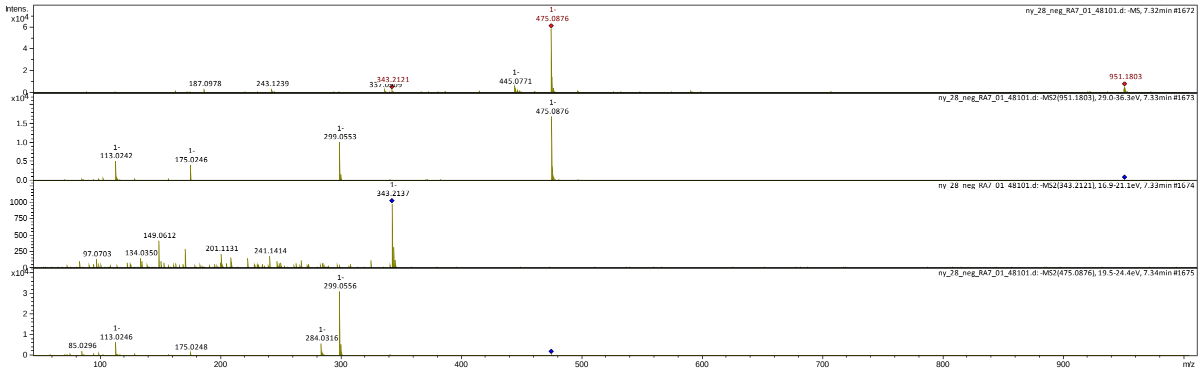

Supplement: Supplementary file 1 [file metabolites-12-00077-s001.zip › metabolites-1534627-supplementary.pdf]
